# Landscape configuration and soil properties shape ant‐mediated seed dispersal in tropical dry forest agricultural landscapes

**DOI:** 10.1111/1365-2656.70313

**Published:** 2026-07-06

**Authors:** Jaiane Maria Silva, Marília Bruzzi Lion, Rafaela Cândido de França, Bráulio Almeida Santos, Helder Farias P. Araujo, José D. Ribeiro‐Neto

**Affiliations:** ^1^ Programa de Pós‐Graduação em Biodiversidade, Centro de Ciências Agrárias Universidade Federal da Paraíba (UFPB) Areia Paraíba Brazil; ^2^ Departamento de Ecologia, Instituto de Ciências Biológicas Universidade Federal de Goiás (UFG), PPG‐ECOEVOL Goiânia Brazil; ^3^ Departamento de Sistemática e Ecologia, Centro de Ciências Exatas e da Natureza Universidade Federal da Paraíba (UFPB) João Pessoa Paraíba Brazil; ^4^ Departamento de Biociências, Centro de Ciências Agrárias Universidade Federal da Paraíba (UFPB) Areia Paraíba Brazil; ^5^ Departamento de Fitotecnia e Ciências Ambientais, Centro de Ciências Agrárias Universidade Federal da Paraíba (UFPB) Areia Paraíba Brazil

**Keywords:** Caatinga, ecosystem service, habitat amount, landscape configuration, landscape heterogeneity, myrmecochory, pH, seed cleaning

## Abstract

1. Ant‐mediated seed dispersal is a key ecological service in seasonally dry tropical forests, contributing to plant regeneration and ecosystem resilience. In human‐modified landscapes, however, the quality and quantity of these interactions may be altered by local and landscape‐scale environmental factors.
2. We investigated how soil properties and landscape aspects influence ant‐mediated seed dispersal in rainfed croplands and natural vegetation remnants within the Caatinga, a dry forest biome in northeastern Brazil. Seed removal and seed cleaning were measured using standardized bait experiments, and interacting ant species were classified by dispersal quality based on body size.
3. Habitat, soil and landscape context jointly shaped ant‐mediated seed dispersal. Croplands supported more high‐quality interactions, while soil pH and sand content influenced interaction quality. At the landscape scale, water availability and fragmentation reduced seed dispersal components, whereas land‐use heterogeneity increased total interactions, likely through small‐bodied, disturbance‐tolerant ants.
4. Distinct ant groups provided different seed dispersal components (cleaning vs removal), shaped by habitat and landscape factors. While plant recruitment and long‐term recovery were not directly assessed, our findings indicate that small‐scale rainfed agricultural landscapes can support functional components of ant‐mediated seed dispersal with potential relevance for dry forest regeneration.

## Graphical abstract

We show that ant‐mediated seed dispersal components respond differently to local soil conditions and landscape structure in Caatinga agricultural mosaics. Low‐intensity rainfed croplands maintained or enhanced seed removal compared to natural remnants, highlighting how small‐scale agriculture can sustain key ecological processes in human‐modified dry forest landscapes.

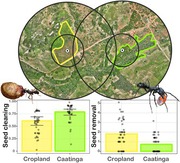

## Resumo

1. A dispersão de sementes mediada por formigas é um serviço ecológico importante em florestas tropicais sazonalmente secas, contribuindo para a regeneração vegetal e a resiliência dos ecossistemas. Em paisagens modificadas por atividades humanas, entretanto, a qualidade e a quantidade dessas interações podem ser alteradas por fatores ambientais locais e em escala de paisagem.
2. Investigamos como propriedades do solo e aspectos da paisagem influenciam a dispersão de sementes mediada por formigas em áreas de agricultura de sequeiro e remanescentes de vegetação natural na Caatinga, um bioma de floresta seca no Nordeste do Brasil. A remoção e a limpeza de sementes foram medidas por meio de experimentos padronizados com iscas, e as espécies de formigas foram classificadas quanto à qualidade da dispersão com base no tamanho corporal.
3. O habitat, o solo e o contexto da paisagem moldaram conjuntamente a dispersão de sementes mediada por formigas. As áreas agrícolas sustentaram mais interações de alta qualidade, enquanto o pH e o teor de areia do solo influenciaram a qualidade das interações. Na escala da paisagem, a disponibilidade de água e a fragmentação reduziram componentes da dispersão de sementes. Já a heterogeneidade de uso da terra aumentou o total de interações, provavelmente por meio de formigas pequenas e tolerantes a distúrbios.
4. Grupos distintos de formigas forneceram diferentes componentes da dispersão de sementes (limpeza vs remoção), moldados por fatores de habitat e paisagem. Embora o recrutamento de plantas e a recuperação de longo prazo não tenham sido avaliados diretamente, nossos resultados indicam que paisagens agrícolas de pequena escala e de sequeiro podem sustentar componentes funcionais da dispersão de sementes mediada por formigas com potencial relevância para a regeneração de florestas secas.

## 1 INTRODUCTION

The conversion of natural habitats into anthropogenic land uses is a major driver of biodiversity loss. This process is associated with disturbances such as habitat loss and fragmentation (Semper‐Pascual et al., 2021; Wilson et al., 2016), edge effects (Coelho et al., 2020; Hurst et al., 2013) and agricultural expansion (Laurance et al., 2014), which lead to substantial changes in environmental conditions. These changes affect a wide range of organisms (Ribeiro‐Neto et al., 2016), fostering declines in taxonomic, functional and phylogenetic diversity (Newbold et al., 2015; Vasconcelos et al., 2024). Such biodiversity losses may promote ecosystem disservices, including the facilitation of invasive species, and compromise key ecosystem functions such as seed dispersal (Rey et al., 2021; Zhao et al., 2024).

Seed dispersal is a mechanism of forest regeneration in abandoned agricultural areas and secondary forest fragments (Reid et al., 2015; Wendt et al., 2022). It includes seed removal from the parent plant, primary dispersal and seed repositioning over the ground, secondary dispersal, largely performed by ants (Prokop et al., 2022; Rico‐Gray & Oliveira, 2007). Many plant species from families such as Euphorbiaceae present elaiosomes, lipid‐rich seed appendages that serve as food rewards for ants in exchange for myrmecochory, a specialized ant‐mediated seed dispersal service (Leal, Neto, et al., 2014; Turner & Frederickson, 2013). Ants, however, also disperse non‐myrmecochorous seeds, which offer arils and fleshy fruit pulp as rewards (Oliveira et al., 2019). Despite this, ant‐mediated seed dispersal is crucial for maintaining and recovering vegetation during succession (Gallegos et al., 2014), enhancing seed germination, reducing sibling competition and density‐dependent mortality (Giladi, 2006).

Ant‐mediated seed dispersal effectiveness depends on both the quantity of seeds removed and the quality of dispersal outcomes (Schupp, 1993; Schupp et al., 2010), including proxies such as seed cleaning (i.e. removal of the attractive food reward). Low seed availability can limit the quantity component of seed dispersal services (de Paula et al., 2023), while functional traits (body size) influence the quality component across different ant species. Larger‐bodied ant species (high‐quality seed dispersers) transport seeds over longer distances, cleaning them inside the nests (Giladi, 2006), whereas smaller‐bodied ant species (low‐quality seed dispersers) clean the seeds locally or transport them over short distances (Andersen & Morrison, 1998; Leal, Neto, et al., 2014). Both ant functional groups (high‐ and low‐quality dispersers) promote seed germination through cleaning, but the transport‐associated benefits for siblings are likely to be reduced in interactions with low‐quality seed dispersers. Consequently, in landscapes dominated by small‐bodied, disturbance‐tolerant ants, seed removal still occurs but results in less effective dispersal. This distinction between removal quantity and dispersal quality has been pointed out as a knowledge gap in recent syntheses of ant–seed interactions (Bona et al., 2023).

Ants' responses to anthropogenic disturbance and forest regeneration are shaped by local and landscape attributes that can affect both components of dispersal effectiveness through changes in ant community composition and behaviour (Burt et al., 2022; Rocha‐Ortega et al., 2017; Schupp, 1993). Locally, soil chemical and physical properties can influence vegetation structure, nest distribution and foraging efficiency, thereby affecting seed‐handling outcomes (Beaumont et al., 2011; Bernadou et al., 2011; Kaspari & Weiser, 1999; Parr et al., 2003; Zhu & Wang, 2019). At the landscape level, habitat amount, edge density and isolation can alter ant communities and dominance structure (Leal et al., 2012; Wirth et al., 2007; Zelikova & Breed, 2008). Recent syntheses indicate that fragmentation can reduce diaspore removal, whereas effects of agricultural landscapes and other land‐use changes on ant‐mediated seed dispersal are context‐dependent (Bona et al., 2023; Cavalcante et al., 2025). Thus, local conditions and landscape structure should be considered jointly as drivers of ant–seed interactions and their outcomes.

The Caatinga is the largest seasonally dry tropical forest in South America and harbours high biodiversity while supporting dense human populations reliant on natural resources (Silva & Barbosa, 2017). Like other seasonally dry tropical forests, it has experienced extensive land‐use change driven by agriculture, logging and grazing, leading to habitat loss, fragmentation and environmental degradation (Araujo et al., 2023). These disturbances have altered vegetation structure and ecosystem functioning, with potential consequences for biodiversity and ecosystem services (Silva et al., 2017). Despite this, Caatinga landscapes, particularly those dominated by smallholder, low‐intensity agriculture, may still support biodiversity and retain ecological functions (Silva et al., 2025). Understanding the extent to which these human‐modified dry forest landscapes continue to deliver ecosystem services to local populations is therefore a pressing scientific challenge and a critical step towards sustainable development.

This study examines how ant‐mediated seed dispersal services respond to environmental variation in subsistence rainfed croplands and Caatinga remnants. We quantified three service components: (i) ant–seed interactions, including the relative contribution of high‐ and low‐quality dispersers; (ii) seed removal; and (iii) seed cleaning. Using a common set of candidate predictors spanning habitat type (croplands vs Caatinga), local edaphic conditions and landscape context, we formulated three predictions. (H1) All service components would be higher in Caatinga remnants than in croplands, reflecting greater habitat complexity and more favourable microclimatic conditions for ant activity and seed handling. (H2) Soil physical and chemical properties (sand content and pH) would explain variation in service components beyond habitat type by influencing ant locomotion, foraging behaviour and functional‐group (high‐ and low‐quality) composition. (H3) Landscapes dominated by anthropogenic land uses and their associated configuration and heterogeneity would influence both the frequency and functional composition of ant–seed interactions, promoting shifts towards low‐quality dispersers and reducing seed dispersal services, whereas higher availability of water bodies was expected to increase ant activity and seed dispersal services.

## 2 MATERIALS AND METHODS

### 2.1 Study site

**FIGURE 1:**
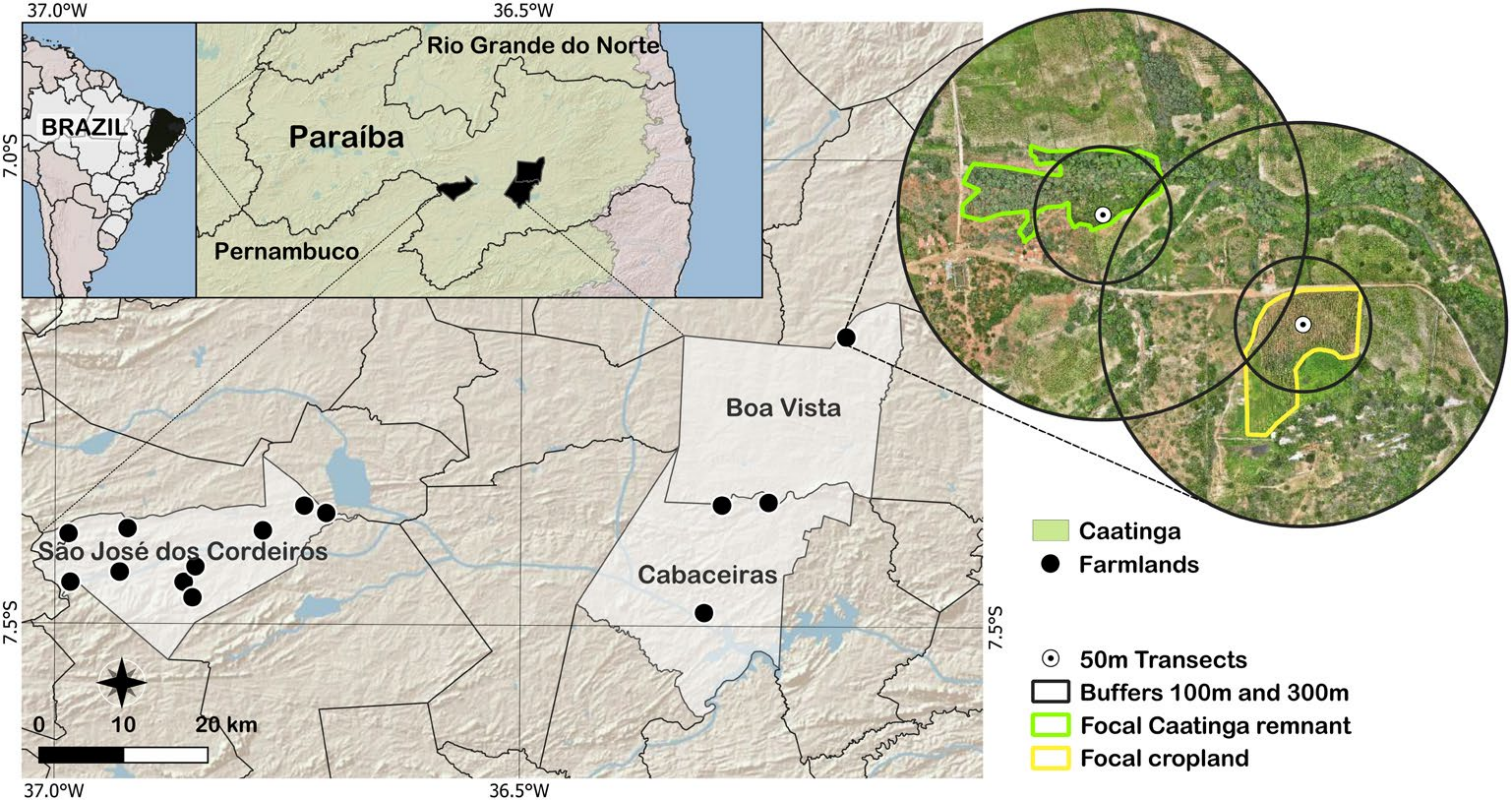
Location of the 14 sampled farmlands in Paraíba, Brazil. The inset shows one focal farm with 50 m transects in cropland (yellow) and adjacent Caatinga forest/woodland (green), and 100 and 300 m buffers.

The study was conducted in the municipalities of São José dos Cordeiros, Cabaceiras and Boa Vista, located in the Cariri region of Paraíba, a semi‐arid area in northeastern Brazil (Figure 1). Annual precipitation ranges from 377 to 573 mm, while the average temperature is 23°C (Diniz et al., 2020). Fourteen smallholder and non‐intensive farms were selected in the region to conduct the seed dispersal experiments (Figure 1). Each farm included both a maize‐bean association cultivated area (cropland) and an adjacent patch of Caatinga remnant. The cultivated areas consisted of small‐scale rainfed agriculture systems, typically occupying up to 2 ha within farms. These systems are managed with family labour and without the use of pesticides. Crops are sown with the first rains of the year—usually between January and March—and harvested by June, after which agricultural activity ceases entirely during the dry season.

Before beginning the experiments, we secured authorisation and consent from all landowners involved. Using the centroid (geographic centre) of each cultivated area as a reference point, we used Google Earth® imagery to visually identify a remaining Caatinga forest patch within a 500 m radius, prioritizing patches with continuous native woody vegetation, minimal visible anthropogenic disturbance and spatial proximity to the cultivated area.

### 2.2 Local and landscape attributes measurements

To characterize the edaphic gradient of the study area, we analysed the chemical and physical composition of the soil in the 14 focal croplands and 14 adjacent Caatinga vegetation remnants. At each site, we established 40 m transects and collected five soil subsamples at 10 m intervals using a cylindrical probe (20 cm length, 7.5 cm diameter). The subsamples from each transect were homogenized into a composite sample and sent to the *Universidade Federal da Paraíba* (UFPB). Soil analyses followed the EMBRAPA methodological manual (EMBRAPA, 2017), quantifying phosphorus (P), potassium (K), sodium (Na), hydrogen + aluminium (H + Al), pH, aluminium (Al), calcium (Ca), magnesium (Mg), sum of exchangeable bases (SB), cation exchange capacity (CEC), sulphur (S) and granulometric fractions (sand, silt and clay). Due to multicollinearity among these variables (Figure S1), we selected pH as an integrative indicator of soil chemical conditions and sand content to represent soil physical structure. pH was chosen because of its central role in regulating nutrient availability and its negative association with soil aluminium concentration, whereas sand content showed the strongest correlations with cation‐related variables, including calcium, magnesium and cation exchange capacity.

The landscape of the region was characterized by drone‐based remote sensing. An overflight at 100 m altitude enabled the creation of an orthomosaic of georeferenced photographs within a 500‐m radius from the centroids of cultivated areas. These images were analysed using QGIS software (QGIS Development Team, v. 3.28), allowing the classification of land use and land cover types at 2.66 cm resolution (pixel size). Initially, we classified land covers into eight categories: cropland, pasture, shrubland, woodland, forest, water bodies, exposed soil (including roads) and civil construction (human settlements). Land‐cover categories were supported by repeated field visits and detailed knowledge of the study farms. Woodland, shrubland and forest classes were distinguished based on vegetation structure, including relative vegetation height, canopy cover and continuity of woody vegetation. Pasture areas were identified by clear indicators of active grazing, such as the presence of livestock (cattle or goats) and well‐defined grazing trails and trampling patterns, whereas areas lacking these features and dominated by woody regrowth were classified as shrubland. These categories were then grouped into four primary land‐use metrics: (1) Caatinga's vegetation remnants, including woodland and forest; (2) farmlands, which include croplands, pastures and shrublands (used for free‐ranging livestock); (3) bare soil, including roads and civil construction; and (4) water bodies. Although shrubland is a natural vegetation type in the Caatinga, recent evidence indicates that its current widespread distribution largely reflects anthropogenically induced forest degradation rather than undisturbed natural conditions (Araujo et al., 2023). In our study area, shrubland patches occurring within farmlands predominantly represent forest‐to‐shrub transitions driven by chronic land use.

Following this classification, we computed landscape metrics for the focal cropland and the focal adjacent Caatinga remnant at two different spatial scales, 100 m and 300 m, because different landscape attributes can affect ant seed dispersal services in divergent ways depending on spatial scale. We selected landscape metrics that captured three key components of landscape structure: (i) habitat loss, represented by habitat amount sensu Fahrig (2013); (ii) habitat configuration, measured as edge density (McGarigal et al., 2023); and (iii) landscape heterogeneity, assessed using the Shannon Diversity Index of anthropogenic land uses (SHDI in McGarigal et al., 2023). Habitat amount (natural cover) was calculated as the percentage of Caatinga remnants within each buffer (100 m or 300 m). Edge density was computed as the total length of edges from Caatinga remnants divided by the buffer area. Edge density was included as a configuration metric because its negative effects on biodiversity are expected to be strongest at intermediate levels of habitat amount (Püttker et al., 2020), which characterize our study landscapes (mean Caatinga cover at 300 m buffers was 26.89 ± 17%, ranging from 0.8% to 77%; see Table S1). SHDI was estimated from five land‐use categories: cropland, pasture, shrubland, bare soil and civil construction. Therefore, shrubland was classified as an anthropogenic land‐cover category of low land‐use intensity, contributing to anthropogenic landscape diversity (anthropic SHDI) but not to the proportion of natural habitat, which was restricted to arboreal Caatinga vegetation. We included the percentage of water bodies within each buffer as an independent variable given its potential influence on local environmental conditions in this seasonally dry system. Raw values of landscape and soil predictors for each farmland are available in Table S1.

### 2.3 Seed dispersal bioassays

Seed dispersal bioassays were conducted along two transects (one in cropland and the other in an adjacent Caatinga remnant, Figure 1), each 50 m in length, featuring six sampling stations. Distance between cropland and Caatinga transects in each farmland varied between 65 m and 480 m. We utilized 10 seeds from three elaiosome‐bearing species in the Euphorbiaceae family that naturally occur in the region: *Croton adamantinus* (small seed size, approximately 0.30 cm in both length and width, approximately globose shape, Oliveira et al., 2023), *Cnidoscolus quercifolius* (intermediate seed size, length 1.14–1.35 cm, width 0.55–0.80 cm, approximately oblong to cylindrical shape, Melo & Sales, 2008) and *Jatropha mollissima* (large seed size, mean length 1.28 cm and mean width 0.82 cm, approximately spherical shape, Araújo et al., 2023). We gathered fruits and extracted seeds, ensuring they were frozen for a few days to maintain their quality before use. The seeds were placed on paper, and the removal criterion was set at 5 cm from the seed mound. Both transects in each site were sampled simultaneously by two researchers along a single day, from 7 to 17 h. Each sampling station was surveyed every 1 h for 5 min. We registered (1) interactions (ants touched seeds with their antennae or walked over them); (2) seed removal, that is, cumulative number of seeds removed during standardized observation periods, with removed seeds replaced; (3) with stereoscopic microscope, proportion of seeds presenting signals of biting by the ants (seed cleaning); and (4) based on size, ants were classified as high‐ (≥5 mm) and low‐quality (<5 mm) seed dispersers. We followed the criteria established by Leal, Neto, et al. (2014) and Oliveira et al. (2019) to classify ants as high‐quality and low‐quality seed dispersers. Ants of medium to large size (≥5.0 mm in body length) are considered high‐quality seed dispersers, while smaller ants (<5.0 mm) are grouped as low‐quality dispersers (Oliveira et al., 2019). However, this classification excludes leafcutter ants (genera *Atta* and *Acromyrmex*), which, despite their size suggesting high‐quality dispersal, carry seeds back to their nests. When these seeds germinate, the ants often cut and kill the seedlings, thus categorizing them as low‐quality dispersers.

Interacting ants were collected, preserved in alcohol and identified whenever possible. We used the identification keys from Baccaro et al. (2015) to classify the ants at the genus and species levels. Ethical approval by an animal ethics committee was not required for this study. Fieldwork and specimen collection were conducted under collection permit no. 60283‐1, issued by the Instituto Chico Mendes de Conservação da Biodiversidade (ICMBio), Brazil. The following variables were used as response variables in subsequent statistical analyses: (1) total interactions, (2) high‐quality interactions percentage, (3) seed removal and (4) seed cleaning percentage.

### 2.4 Statistical analysis

For each response variable (total interactions, high‐quality interaction percentage, seed removal and seed cleaning percentage), we fitted Generalized Linear Mixed Models (GLMMs, Bates et al., 2015), including farmland (the 14 sampled farms) and seed plant species (*Cnidoscolus quercifolius*, *Croton adamantinus* and *Jatropha mollissima*) as random effects, with plant species nested within farmland, to account for non‐independence of observations and species‐specific variability. Additive models included seven fixed‐effect predictors: (i) local attributes, (1) *habitat type* (cropland vs Caatinga remnant), (2) *soil sand content* (g/kg) and (3) *soil pH*; and (ii) landscape attributes at their best‐fitting scale, (4) *percentage of Caatinga*, (5) *edge density* (m/ha), (6) *percentage of water bodies* and (7) the diversity index of anthropogenic land use—*SHDI anthropic*.

To assess the scale effect of each landscape metric, we tested each of these metrics individually as a fixed effect at two spatial scales (100 m and 300 m). The optimal scale for each metric was determined by the lowest Akaike's Information Criterion (AIC) value (Table S2). After scale selection, all models were then fitted within a single hierarchical framework containing two random effects (farmland and seed plant species) and seven fixed effects, each evaluated at its best‐fitting scale (lowest AIC). To compare the relative strength of fixed effect predictors, all continuous predictors were *Z*‐scaled before running the models. Since high‐quality interaction and seed cleaning were percentage data, we used a binomial error distribution for these models. We initially modelled seed removal and total interactions using Poisson error distributions, as expected for count data, but these models showed convergence issues and poor fit. We therefore applied square‐root (seed removal) and log (total interactions) transformations and fitted Gaussian models, a common approach in ecological studies when count models perform poorly. We used the function ‘simulateResiduals’ from the ‘DHARMa’ R package to assess residual distribution, dispersion, absence of outliers and the absence of spatial autocorrelation (Hartig, 2022). Although some overlap among buffers was possible given the spatial configuration of sites, tests for spatial autocorrelation in model residuals indicated no significant spatial dependence, supporting the assumption of independence among transects. We assessed multicollinearity among predictors using variance inflation factors (VIF). Values were low for landscape variables (all <3), while habitat type and soil pH showed slightly higher values (<4), still below commonly accepted thresholds.

Based on these full models, we performed model selection using AIC corrected for small sample sizes (AICc) to rank all models in order of increasing AICc, using the MuMIn R package (Barton, 2024). Models with ΔAICc ≤2 were retained as the best models. We then conducted model averaging to estimate the averaged coefficient for each predictor and its relative importance (sum of weights). Predictors consistently present in all selected models (i.e. with maximum relative importance) were plotted for interpretation and discussion.

## 3 RESULTS

We recorded a total of 615 ant–seed interactions. In total, 40 ant species from 13 genera and 5 subfamilies were observed feeding on elaiosomes, inspecting or transporting seeds (Table S3). Of these, eight species were classified as high‐quality seed dispersers, accounting for 28% of the interactions, while the remaining 32 species were considered low‐quality dispersers and accounted for 72% of the interactions.

Out of the 4203 seeds offered during the experiments, 273 were removed, corresponding to an overall proportion of seeds removed of 6.5%. Seed removal was higher in croplands, where 220 seeds were removed (80.6% of all removals), compared to Caatinga remnants, which accounted for 53 removals (19.4%). Seven ant species dominated interaction records, encompassing both seed removal and cleaning events. Among these, five species were classified as providing low‐quality interactions: *Pheidole* sp. (75 interactions), *Dorymyrmex thoracicus* (56), *Dolichoderus doloniger* (46), *Acromyrmex rugosus* (45) and *Pheidole* sp. 4 (42). Whereas two species were classified as high‐quality interaction providers: *Camponotus crassus* (60 interactions) and *Camponotus balzani* (43). These species disproportionately contributed to seed removal and cleaning processes across habitats.

From the total interactions observed, *Jatropha mollissima* (the most interactive plant species) presented 2.4 times more interactions than *Croton adamantinus* (the least interactive plant species; Table S4). Cropland plots had 8% more interactions than plots in the adjacent forest/woodland patch (Table S4).

**FIGURE 2:**
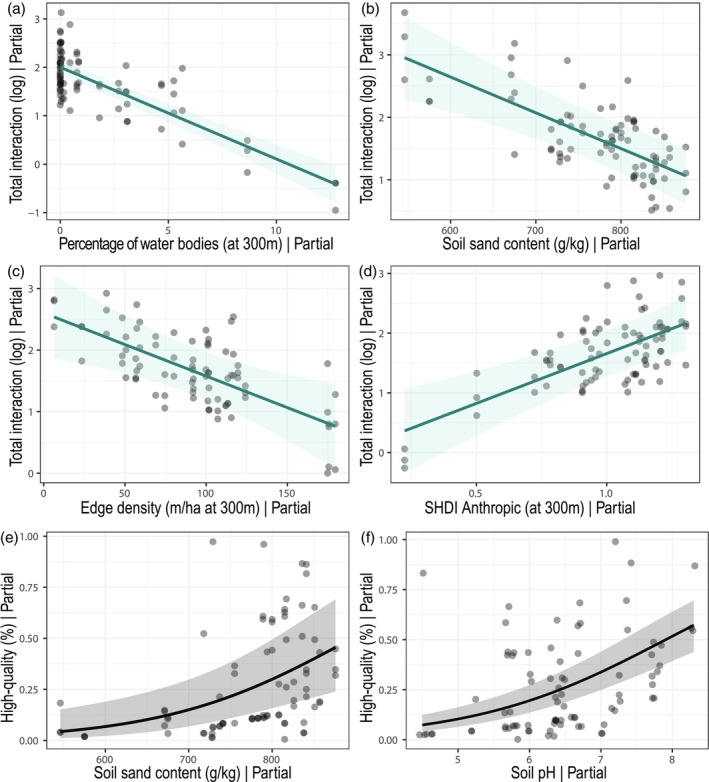
Partial effects of environmental predictors on (a–d) log‐transformed total ant–seed interactions and (e, f) the proportion of high‐quality interactions. Predictors include (a) water bodies (%) at 300 m, (b, e) soil sand content (g/kg), (c) edge density (m/ha) at 300 m, (d) Shannon diversity of anthropic land uses at 300 m and (f) soil pH. Total interactions are shown in green and high‐quality interactions in black. Partial effects represent predictor effects holding other variables constant.

Only one model predicting total interactions was retained by the model selection approach, based on ΔAICc ≤2 (Table 1). This model encompassed four predictor variables: percentage of water bodies (300 m scale), soil sand content and edge density (300 m scale), exerting negative effects and anthropogenic use diversity index (300 m scale), which had a positive effect on total interaction (Table 1; Figure 2a–d).

**TABLE 1:** Results of generalized linear mixed models assessing the effects of local and landscape predictors on four response variables: Total interactions, percentage of high‐quality interactions, seed removal and seed cleaning.

	Coefficient ± SE	*p* value	R^2^m	R^2^c
Total interaction			0.31	0.78
Soil sand content	−0.453 ± 0.094	**<0.001**		
Edge density (300 m)	−0.431 ± 0.137	**0.003**		
Percentage of water bodies (300 m)	−0.578 ± 0.112	**<0.001**		
SHDI anthropic (300 m)	0.401 ± 0.093	**<0.001**		
High‐quality interaction (%)				
Model 1			0.25	0.89
Soil sand content	0.97 ± 0.24	**<0.001**		
Soil pH	0.81 ± 0.19	**<0.001**		
Percentage of water bodies (300 m)	0.55 ± 0.32	0.09		
SHDI anthropic (100 m)	−0.55 ± 0.27	0.04		
Model 2			0.23	0.89
Soil sand content	0.89 ± 0.24	**<0.001**		
Soil pH	0.81 ± 0.19	**<0.001**		
SHDI anthropic (100 m)	−0.46 ± 0.27	0.09		
Model 3			0.22	0.87
Soil sand content	0.7 ± 0.2	**<0.001**		
Soil pH	0.67 ± 0.16	**<0.001**		
Model 4			0.24	0.86
Soil sand content	0.72 ± 0.2	**<0.001**		
Soil pH	0.65 ± 0.16	**<0.001**		
Percentage of water bodies (300 m)	0.36 ± 0.29	0.21		
Averaged Model			Relative importance (sum of weights)	
Soil sand content	0.852 ± 0.256	**0.001**	1	
Soil pH	0.758 ± 0.196	**<0.001**	1	
Percentage of water bodies (300 m)	0.238 ± 0.337	0.479	0.48	
SHDI anthropic (100 m)	−0.327 ± 0.329	0.32	0.65	
Seed removal				
Habitat type (Caatinga remnant)	−1.082 ± 0.24	**<0.001**	0.18	0.27
Seed cleaning (%)				
Model 1			0.14	0.99
Habitat type (Caatinga remnant)	0.95 ± 0.28	**<0.001**		
Edge density (300 m)	−1.3 ± 0.32	**<0.001**		
Soil pH	1.39 ± 0.19	**<0.001**		
Percentage of water bodies (300 m)	−0.5 ± 0.16	**<0.001**		
SHDI anthropic (300 m)	0.42 ± 0.17	**0.01**		
Percentage of Caatinga (300 m)	0.49 ± 0.29	0.09		
Model 2			0.12	0.99
Habitat type (Caatinga remnant)	0.97 ± 0.27	**<0.001**		
Edge density (300 m)	−0.94 ± 0.24	**<0.001**		
Soil pH	1.34 ± 0.18	**<0.001**		
Percentage of water bodies (300 m)	−0.39 ± 0.14	**0.01**		
SHDI anthropic (300 m)	0.27 ± 0.14	0.054		
Model 3			0.12	0.99
Habitat type (Caatinga remnant)	0.87 ± 0.26	**<0.001**		
Edge Density (300 m)	−1 ± 0.25	**<0.001**		
Soil pH	1.32 ± 0.18	**<0.001**		
Percentage of water bodies (300 m)	−0.23 ± 0.11	**0.04**		
Averaged Model			Relative importance (sum of weights)	
Habitat type (Caatinga remnant)	0.939 ± 0.28	**0.001**	1	
Edge density (300 m)	−1.105 ± 0.329	**0.001**	1	
Soil pH	1.358 ± 0.19	**<0.001**	1	
Percentage of water bodies (300 m)	−0.401 ± 0.177	**0.024**	1	
SHDI anthropic (300 m)	0.271 ± 0.211	0.197	0.78	
Percentage of Caatinga (300 m)	0.205 ± 0.308	0.506	0.42	

*Note*: We report coefficients (±SE) and *p* values for predictors retained in the best‐supported models (ΔAICc ≤2). When multiple models were supported, results include individual models and model‐averaged estimates with predictor relative importance (sum of Akaike weights). Marginal (*R* ^2^m) and conditional (*R* ^2^c) coefficients of determination indicate variance explained by fixed effects alone and by fixed plus random effects. Landscape predictors are shown at the best‐fitting spatial scale (100 or 300 m). Random effects were farmland and seed plant species (nested within farmland). Continuous predictors were *Z*‐standardized. Significant effects (*p* < 0.05) are shown in bold.

For the proportion of high‐quality interactions, four models were considered equally probable (ΔAICc ≤2; Table 1). These models included four predictor variables: two at the local scale—soil sand content and pH—and two at the landscape scale—percentage of water bodies (300 m scale) and diversity of anthropogenic land use (100 m scale). An increase in soil sand content and pH was associated with a higher proportion of high‐quality seed disperser interactions (Table 1; Figure 2e,f). The percentage of water bodies also had a positive effect, though this was not statistically significant in the averaged model (Table 1). Conversely, an increase in the anthropogenic SHDI was associated with a decrease in the proportion of high‐quality seed disperser interactions, but this effect was also not significant in the averaged model (Table 1).

**FIGURE 3:**
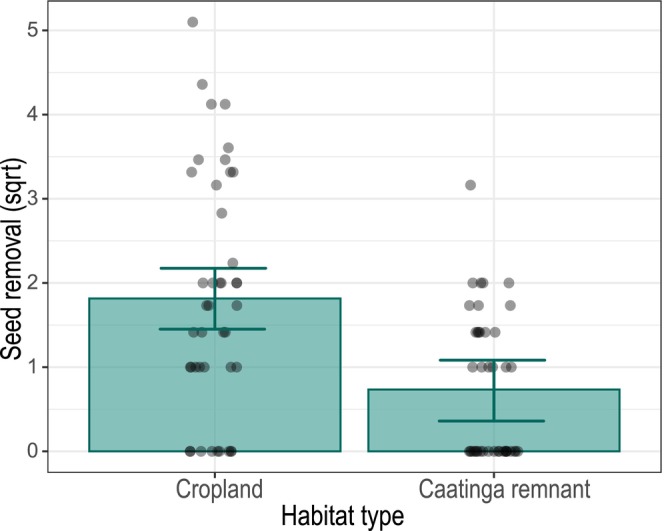
Effects of habitat type on seed removal by ants. Bars show mean square root‐transformed seed removal in cropland and Caatinga remnants, with standard errors. Points represent partial residuals from mixed models with habitat type as fixed effect and seed species nested within farmland as random effects.

Regarding seed removal, only one model was selected, in which habitat type was included as a predictor (Table 1; Figure 3). On average, seed removal in cropland sites was more than twice as high as in natural remnants (Figure 3).

**FIGURE 4:**
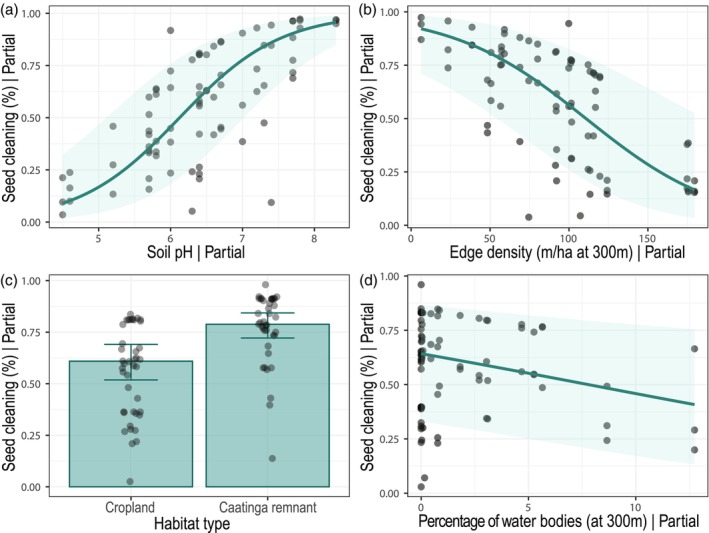
Partial effects of environmental predictors on the proportion of seeds cleaned by ants: (a) soil pH, (b) edge density (m/ha) at 300 m, (c) habitat type and (d) water cover (%) at 300 m. In panel (c), bars indicate mean values per habitat with standard errors. Partial effects represent predictor effects holding other variables constant.

For seed cleaning percentage, three models were selected (Table 1; Figure 4a–d). Across these models, six predictors were included, four of which were consistently present in all selected models (those with ΔAICc ≤2) and had significant coefficients (*p* < 0.05): habitat type, edge density (300 m scale), soil pH and percentage of water bodies (300 m scale). The percentage of water bodies and edge density had a negative effect on seed cleaning percentage, whereas soil pH had a positive effect, and seed cleaning was more frequent in natural remnants (Figure 4). Two additional predictors—anthropogenic SHDI and percentage of natural cover (both at the 300 m scale)—were also selected but had lower relative importance (Table 1).

## 4 DISCUSSION

Our study examined how local and landscape‐scale attributes shape ant‐mediated seed dispersal services in croplands and Caatinga remnants, explicitly testing three hypotheses. Contrary to our first hypothesis (H1), which predicted higher levels of ant‐mediated services in Caatinga remnants, seed removal was higher in croplands, whereas seed cleaning was more frequent in natural remnants, indicating that different components of the dispersal services respond differently to habitat type. Consistent with our second hypothesis (H2), local edaphic conditions, particularly soil pH and sand content, strongly influenced interaction quality, favouring a higher proportion of interactions performed by large‐bodied, high‐quality dispersers under specific soil conditions. Finally, we found partial support for our third hypothesis (H3): landscapes dominated by anthropogenic land uses and their associated configuration and heterogeneity influenced both the frequency and functional composition of ant–seed interactions, promoting shifts towards low‐quality dispersers and reducing seed dispersal services, whereas the effect of water availability was opposite to our initial expectation. Together, these results highlight that ant‐mediated seed dispersal is structured by the combined but independent effects of local soil conditions and landscape context, with important consequences for the balance between high‐ and low‐quality dispersers across human‐modified dry forest landscapes.

Previous studies conducted in the Caatinga have consistently shown that ant‐mediated seed dispersal services, particularly those provided by high‐quality dispersers, tend to decline under increasing anthropogenic disturbance, including chronic land use, aridity intensification and habitat degradation (Leal, Andersen, & Leal, 2014; Oliveira et al., 2019). Based on this evidence, we expected higher levels of seed dispersal services in Caatinga remnants than in croplands. However, the contrasting pattern observed here suggests that small‐scale, rainfed agriculture systems may not conform to the disturbance gradients typically examined in previous studies. These findings indicate that such traditional agricultural systems may function as low‐impact systems under the conditions studied. This interpretation is consistent with studies showing that low‐intensity, smallholder agricultural systems can maintain biodiversity and support ecosystem services within heterogeneous landscapes (Perfecto & Vandermeer, 2010; Tscharntke et al., 2012). Under these conditions, croplands may increase habitat openness and thermal heterogeneity while remaining functionally connected to native vegetation, favouring large‐bodied, effective ant dispersers and sustaining seed dispersal services.

Local edaphic conditions shape ant community composition and behaviour, with direct consequences for seed dispersal services (Mugnai et al., 2021; Silva et al., 2019). In our study, soil texture appeared to act as an environmental filter on ant functional groups, helping shift the balance between low‐ and high‐quality dispersers. In the Caatinga, clayey soils are often associated with rocky substrates that create structurally complex surfaces, favouring interstitial foraging by small‐bodied ants (Kaspari & Weiser, 1999; Parr et al., 2003). By contrast, sandy soils provide flatter surfaces that facilitate the movement of larger ants, promoting more effective dispersal. Additionally, soil chemistry, particularly pH, is associated with nutrient availability and plant performance (Brady & Weil, 2016; Viani et al., 2014), affecting Caatinga species richness and productivity (Maciel et al., 2024). Similarly, pH may influence seed‐handling outcomes through its association with nutrient availability, plant performance and habitat structure. Thus, soil texture and pH do not simply affect interaction frequency but reshape the functional composition of interacting ants and their behaviour, increasing dispersal effectiveness, despite fewer total interactions. Such effects, mediated by the filtering of ant functional traits through soil–vegetation–biota linkages, have been reported in dry forest systems (Apgaua et al., 2015; Maia et al., 2020). However, higher contributions of high‐quality dispersers do not necessarily result in greater seedling establishment, as outcomes depend on local abiotic and biotic conditions and disturbance regimes (Giladi, 2006).

Landscape structure emerged as a driver of ant–seed interactions, highlighting the importance of habitat configuration over habitat amount in these systems. Although land‐use change is widely recognized as a major driver of biodiversity loss and ecosystem service degradation (Foley et al., 2005; Rodríguez‐Echeverry et al., 2018), habitat amount was not a key predictor in our models. Instead, habitat configuration appeared more informative, consistent with the idea that configuration can be especially relevant in landscapes with intermediate habitat cover. This finding contributes to the ongoing debate in landscape ecology regarding the relative importance of habitat amount versus configuration (Fahrig et al., 2019; Fletcher et al., 2018), supporting the idea that configuration plays a more prominent role in landscapes with intermediate levels of habitat cover (Püttker et al., 2020), such as those studied here. The unexpected negative association with water bodies may reflect greater availability of alternative food resources, such as insects, carrion (Bonfoey et al., 2023), extra‐floral nectar (da Cruz Rocha et al., 2019) and honeydew (Kansman et al., 2021), potentially reducing seed attractiveness to small‐bodied, low‐quality dispersers that often act as predators, scavengers or seed predators rather than effective dispersers (Andersen & Morrison, 1998; Karnish, 2024). The positive association with landscape heterogeneity likely reflects increased activity of small‐bodied, generalist ants, which are often favoured in heterogeneous anthropogenic landscapes (Andersen, 2019; Andersen et al., 2000), suggesting that higher interaction rates primarily indicate shifts in community composition towards low‐quality seed dispersers. Finally, the stronger effects detected at the 300 m scale indicate that ant‐mediated seed dispersal responds to landscape structure at spatial extents consistent with ant foraging ranges and movement across habitat boundaries, rather than being driven solely by local conditions.

Our results also indicate that different species are responsible for seed cleaning and seed removal. Small ants, classified as low‐quality dispersers, typically consume elaiosomes without moving the seeds (Andersen & Morrison, 1998), while larger ants are more effective at promoting seed removal (Karnish, 2024). This is further supported by the effects of habitat type on these functions: while croplands promoted seed removal, natural remnants favoured seed cleaning (after accounting for other fixed effects). Vegetation in remaining Caatinga patches is more complex in structure than vegetation structure in croplands, and, consequently, it produces higher amounts of leaf litter and provides a more shadowed soil surface, reducing temperature, if compared to croplands. Both attributes are known to affect ants: higher leaf litter should favour small ants (Kaspari & Weiser, 1999) and a shadowed and colder soil surface can be differentially used by ants (Silva et al., 2019). Thus, in remaining Caatinga patches, small ants may be favoured by their ability to forage interstitially through leaf litter and, under cooler and shadier conditions, spend more time handling seeds and consuming elaiosomes in situ. On the other hand, the flat soil surface of croplands may favour large‐bodied ants and, to escape the high temperature, ants may grab the whole seed, transport them into the nest and only then get the elaiosomes, discharging the cleaned seed in refuse dumps. These findings suggest that high‐quality dispersers are more prone to move seeds in Caatinga croplands, potentially contributing to early seed‐dispersal processes relevant to regeneration in abandoned agricultural areas, although subsequent recruitment was not assessed.

## 5 CONCLUSIONS

Our study highlights that small‐scale, low‐intensity agricultural matrices can retain functional components of ant‐mediated seed dispersal in tropical dry forest landscapes. In these rainfed systems, characterized by low external inputs, family labour and strong seasonal dynamics, agricultural areas and adjacent native vegetation appear to provide complementary conditions for different components of the dispersal process. This suggests that the ecological value of agricultural matrices depends on the interplay among habitat type, local soil conditions, landscape configuration and the functional composition of interacting ant assemblages. By considering seed cleaning and seed removal together with the relative contribution of high‐ and low‐quality dispersers, our study emphasizes the importance of integrating quantity and quality components of dispersal effectiveness when assessing ecosystem services in human‐modified dry forests. These findings reinforce the importance of maintaining adjacent native vegetation and heterogeneous, low‐intensity agricultural landscapes to conserve ecological functions relevant to dry forest regeneration, while highlighting the need for future research to assess how differences in ant‐mediated seed dispersal services affect plant recruitment and long‐term ecosystem recovery.

## AUTHOR CONTRIBUTIONS

José D. Ribeiro‐Neto, Bráulio Almeida Santos and Helder Farias P. Araujo conceived the ideas and designed the methodology. Jaiane Maria Silva collected the data, and Marília Bruzzi Lion analysed the data. Jaiane Maria Silva and Rafaela Cândido de França wrote the first draft of the manuscript. Marília Bruzzi Lion and José D. Ribeiro‐Neto led the writing of the final manuscript. All authors contributed critically to the drafts and approved the final version for publication.

## CONFLICT OF INTEREST STATEMENT

All authors declare that they have no conflicts of interest to disclose.

## STATEMENT ON INCLUSION

All authors are Brazilian researchers based in different regions of the country, bringing together diverse local perspectives and expertise. The study was conducted in close collaboration with smallholder farming families who own and manage the lands studied. Throughout the fieldwork, the authors engaged with these stakeholders to discuss ecological processes relevant to their daily lives, including seed dispersal and pollination, and how these processes underpin ecosystem services that contribute to the regeneration and sustainability of Caatinga landscapes. As part of our outreach efforts, each participating family received a framed aerial image of their property, generated from high‐resolution drone orthophotos. These images were very well received—often displayed proudly in the homes—and served as a valuable entry point for discussing ecological concepts. Through these interactions, local participants were introduced to the idea of ecosystem services, fostering dialogue between scientific knowledge and lived experience in the region.

## Supporting information


**Table S1.** Raw values of landscape and soil predictors, across landscapes (14 Farmlands) and between habitat types (croplands and Caatinga remnants).
**Table S2.** Akaike's Information Criterion (AIC) for models predicting the four response variables modelled at both scales (100 and 300 m): total seed–ant interactions, high‐quality interaction, seed removal and seed cleaning. The optimal scale was determined by the lowest AIC value and is highlighted.
**Table S3.** Sampled species according to it classification related to seed dispersion quality. High‐quality are species with body size ≥5 mm, while low‐quality species are below 5 mm in size.
**Table S4.** Mean values (±95% confidence intervals) of biological response variables for three plant species (*Cnidoscolus quercifolius*, *Croton adamantinus* and *Jatropha mollissima*) across two habitat types (cropland and natural Caatinga remnant). The variables include total interactions, high‐ and low‐quality interactions, seed removal and percentage of seed cleaning. Farmland indicates the number of landscapes where experiments were conducted for each habitat–plant combination.
**Figure S1.** Pearson correlation matrix of soil chemical and physical variables measured across 14 subsistence rainfed croplands and 14 adjacent Caatinga vegetation remnants. Variables include soil pH, aluminium (Al^3+^), hydrogen + aluminium (H + Al), phosphorus (P), nitrogen (N), sulphur (S), exchangeable bases (Ca^2+^, Mg^2+^, K^+^, Na^+^), sum of bases (SB), cation exchange capacity (CEC) and granulometric fractions (sand, silt and clay). Colours represent the strength and direction of correlations (blue = positive; red = negative), with correlation coefficients shown in each cell.

## Data Availability

All data and scripts used in the analyses are available at https://doi.org/10.17605/OSF.IO/HV2WF (Silva et al., 2026).
