# Supplementary material for: Landscape configuration and soil properties shape ant‐mediated seed dispersal in tropical dry forest agricultural landscapes
Source: J Anim Ecol. 2026 Jul 6;95(8):1476–87. doi: 10.1111/1365-2656.70313 (PMC13439585; doi:10.1111/1365-2656.70313)
Supplement: Supplementary file 1 [file JANE-95-1476-s001.docx]

**Landscape configuration and soil properties shape ant-mediated seed dispersal in tropical dry forest agricultural landscapes**

Supplementary file

**Table S1.** Raw values of landscape and soil predictors, across landscapes (14 Farmlands) and between habitat types (croplands and Caatinga remnants).

| **Farmland** | **Habitat** | **Caatinga (%) 100m** | **Caatinga (%) 300m** | **Water bodies (%) 100m** | **Water bodies (%) 300m** | **Edge density (m/ha) 100m** | **Edge density (m/ha) 300m** | **SHDI anthropic 100m** | **SHDI anthropic 300m** | **Soil sand content (g/kg)** | **Soil pH** |
| --- | --- | --- | --- | --- | --- | --- | --- | --- | --- | --- | --- |
| Daniel | Caatinga | 60.29 | 31.86 | 4.94 | 3.05 | 119.62 | 101.46 | 0.3 | 0.82 | 790 | 4.5 |
| Daniel | Cropland | 14.37 | 30.96 | 8.85 | 3.1 | 144.48 | 101.71 | 0.26 | 1.11 | 575 | 6.6 |
| Ema | Caatinga | 61.08 | 19.4 | 0 | 0 | 256.78 | 119.57 | 0.13 | 0.78 | 800 | 6.7 |
| Ema | Cropland | 16.45 | 18.33 | 0 | 0 | 114.01 | 124.17 | 0.89 | 1.21 | 841 | 7.7 |
| Francisco | Caatinga | 91.14 | 38.8 | 0 | 0.45 | 71.83 | 174.83 | 0 | 1.2 | 675 | 6.4 |
| Francisco | Cropland | 24 | 41.98 | 0 | 0.04 | 149.72 | 179.51 | 0.49 | 1.13 | 841 | 5.7 |
| Guilherme | Caatinga | 0 | 0.8 | 0 | 0.85 | 0 | 6.38 | 0.22 | 0.77 | 794 | 5.2 |
| Guilherme | Cropland | 0 | 3.01 | 4.48 | 12.74 | 0 | 23.39 | 1.32 | 1.23 | 729 | 7.2 |
| Janga | Caatinga | 32.06 | 20.93 | 0 | 0.04 | 151.52 | 116.77 | 0.89 | 1 | 808 | 5.7 |
| Janga | Cropland | 21.01 | 17.18 | 0 | 0 | 63.52 | 74.53 | 0.68 | 1.08 | 836 | 7.4 |
| Lapa | Caatinga | 86.28 | 54.48 | 0 | 0 | 70.18 | 99.86 | 0 | 1.12 | 755 | 6 |
| Lapa | Cropland | 35.76 | 36.92 | 0 | 0 | 240 | 92.15 | 0.52 | 1.15 | 826 | 7.3 |
| Luciano | Caatinga | 47.32 | 23.95 | 0 | 0 | 126.5 | 80.15 | 0.78 | 0.5 | 776 | 4.6 |
| Luciano | Cropland | 0.33 | 29.12 | 0 | 0 | 8.16 | 91.46 | 0.92 | 0.72 | 851 | 6.3 |
| Nelquides | Caatinga | 51.64 | 22.69 | 1.52 | 1.82 | 215.83 | 175.57 | 0.77 | 1.3 | 783 | 6.4 |
| Nelquides | Cropland | 0 | 12.93 | 12.44 | 5.65 | 0 | 115.51 | 0.88 | 1.1 | 737 | 7.7 |
| Nivaldo | Caatinga | 37.43 | 38.44 | 0 | 0.77 | 89.39 | 48.34 | 1.32 | 0.96 | 817 | 6.4 |
| Nivaldo | Cropland | 35.15 | 43.16 | 0 | 0.77 | 79.74 | 59.11 | 1.16 | 0.97 | 876 | 7.8 |
| Rico | Caatinga | 0 | 19.18 | 0 | 4.68 | 0 | 101.1 | 0.82 | 0.91 | 815 | 5.8 |
| Rico | Cropland | 0 | 10.95 | 20.85 | 8.66 | 0 | 50.59 | 0.98 | 1.19 | 789 | 5.7 |
| Salambaia | Caatinga | 56.51 | 77.73 | 0 | 0.16 | 174.26 | 107.19 | 0 | 0.22 | 728 | 6.3 |
| Salambaia | Cropland | 17.26 | 38.83 | 2.61 | 5.26 | 118.89 | 38.6 | 0.51 | 1.29 | 671 | 6.5 |
| Sitiobravo | Caatinga | 21.15 | 6.24 | 0 | 0 | 172.39 | 56.67 | 1.07 | 0.91 | 718 | 6.7 |
| Sitiobravo | Cropland | 22.31 | 6.36 | 0 | 0.01 | 172.64 | 57.25 | 1.05 | 0.92 | 546 | 8.3 |
| Vitoria | Caatinga | 86.68 | 45.97 | 1.2 | 2.7 | 90.91 | 91.9 | 0 | 0.94 | 741 | 5.8 |
| Vitoria | Cropland | 14.56 | 18.62 | 0 | 0.08 | 68.35 | 68.9 | 0.86 | 1.07 | 857 | 6.4 |
| Vovitoria | Caatinga | 59.78 | 25.82 | 0 | 0 | 213.89 | 112.12 | 1.09 | 1.15 | 815 | 6 |
| Vovitoria | Cropland | 0 | 18.2 | 0 | 0.03 | 0 | 113.33 | 0.93 | 1.21 | 838 | 7 |
|  |  | mean (SD) range | mean (SD) range | mean (SD) range | mean (SD) range | mean (SD) range | mean (SD) range | mean (SD) range | mean (SD) range | mean (SD) range | mean (SD) range |
| *All landscapes* | Caatinga | 49.38 (29.14)  0 - 91.14 | 30.45 (19.84)  0.80 - 77.73 | 0.55 (1.36)  0 - 4.94 | 1.04 (1.47)  0 - 4.68 | 125.22 (77.68)  0 - 256.78 | 99.42 (44.67)  6.38 - 175.57 | 0.53 (0.48)  0 - 1.32 | 0.90 (0.28)  0.22 - 1.30 | 772.50 (43.19)  675.00 - 817.00 | 5.89 (0.70)  4.50 - 6.70 |
| *All landscapes* | Cropland | 14.37 (12.79)  0 - 35.76 | 23.33 (13.39)  3.01 - 43.16 | 3.52 (6.33)  0 - 20.85 | 2.59 (4.05)  0 - 12.74 | 82.82 (76.92)  0 - 240.00 | 85.02 (40.68)  23.39 - 179.51 | 0.82 (0.29)  0.26 - 1.32 | 1.10 (0.15)  0.72 - 1.29 | 772.36 (106.92)  546.00 - 876.00 | 6.97 (0.80)  5.70 - 8.30 |

**Table S2.** Akaike’s Information Criterion (AIC) for models predicting the four response variables modelled at both scales (100 and 300m): total seed-ant interactions, high-quality interaction, seed removal, and seed cleaning. The optimal scale was determined by the lowest AIC value and is highlighted.

| ***Total seed-ant interactions*** | ***AIC*** |
| --- | --- |
| Percentage of Caatinga 100m | 255.4 |
| Percentage of Caatinga 300m | 249.5 |
| Percentage of water bodies 100m | 251.2 |
| Percentage of water bodies 300m | 248.4 |
| Edge density 100m | 257.2 |
| Edge density 300m | 252.6 |
| SHDI anthropic 100m | 246.6 |
| SHDI anthropic 300m | 242.2 |
| ***High-quality interaction*** | ***AIC*** |
| Percentage of Caatinga 100m | 285.0 |
| Percentage of Caatinga 300m | 293.7 |
| Percentage of water bodies 100m | 293.2 |
| Percentage of water bodies 300m | 292.2 |
| Edge density 100m | 290.5 |
| Edge density 300m | 291.0 |
| SHDI anthropic 100m | 291.7 |
| SHDI anthropic 300m | 292.2 |
| ***Seed removal*** | ***AIC*** |
| Percentage of Caatinga 100m | 287.6 |
| Percentage of Caatinga 300m | 290.3 |
| Percentage of water bodies 100m | 286.8 |
| Percentage of water bodies 300m | 290 |
| Edge density 100m | 292.6 |
| Edge density 300m | 293 |
| SHDI anthropic 100m | 291.9 |
| SHDI anthropic 300m | 286.8 |
| ***Seed cleaning*** | ***AIC*** |
| Percentage of Caatinga 100m | 659.4 |
| Percentage of Caatinga 300m | 649.5 |
| Percentage of water bodies 100m | 658.3 |
| Percentage of water bodies 300m | 653 |
| Edge density 100m | 646.2 |
| Edge density 300m | 644.6 |
| SHDI anthropic 100m | 657 |
| SHDI anthropic 300m | 645.7 |

Table S3. Sampled species according to it classification related to seed dispersion quality. High-quality are species with body size ≥5mm, while low-quality species are below 5mm in size.

| Low-quality (32) | High-quality (8) |
| --- | --- |
| **Dolichoderinae** | **Ectatomminae** |
| *Dolichoderus doloniger* | *Ectatomma brunneum* |
| *Dorymyrmex thoracicus* | *Ectatomma muticum* |
| *Dolichoderus* sp | **Formicinae** |
| *Dolichoderus* sp1 | *Camponotus balzani* |
| *Dolichoderus* sp2 | *Camponotus crassus* |
| *Dolichoderus* sp3 | *Camponotus rufipes* |
| **Formicinae** | *Camponotus* sp1 |
| *Brachymyrmex aphicola* | *Camponotus* sp2 |
| **Myrmicinae** | **Ponerinae** |
| *Acromyrmex rugosus* | *Dinoponera quadriceps* |
| *Atta sexdens* |  |
| *Cephalotes adolphi* |  |
| *Cephalotes inca* |  |
| *Crematogaster* sp |  |
| *Crematogaster jardinero* |  |
| *Crematogaster* sp1 |  |
| *Crematogaster* sp2 |  |
| *Pheidole fracticeps* |  |
| *Pheidole* sp |  |
| *Pheidole radoszkowskii* |  |
| *Pheidole* sp1 |  |
| *Pheidole* sp2 |  |
| *Pheidole* sp3 |  |
| *Pheidole* sp4 |  |
| *Pheidole* sp5 |  |
| *Solenopsis* sp |  |
| *Solenopsis saevissima* |  |
| *Solenopsis* sp1 |  |
| *Solenopsis* sp2 |  |
| *Solenopsis* sp3 |  |
| *Solenopsis* sp4 |  |
| *Solenopsis tridens* |  |
| *Solenopsis virulens* |  |
| *Tetramorium* sp |  |

**Table S4.** Mean values (± 95% confidence intervals) of biological response variables for three plant species (*Cnidoscolus quercifolius*, *Croton adamantinus*, and *Jatropha mollissima*) across two habitat types (cropland and natural Caatinga remnant). The variables include total interactions, high- and low-quality interactions, seed removal, and percentage of seed cleaning. Farmland indicates the number of landscapes where experiments were conducted for each habitat–plant combination.

| Biological variable | Plant species | Habitat | Farmlands | Means | 95% CI |
| --- | --- | --- | --- | --- | --- |
| Total seed-ant interactions | *Cnidoscolus quercifolius* | Cropland | 14 | 5.50 | 3.41 |
|  |  | Caatinga remnant | 14 | 5.36 | 2.87 |
|  | *Croton adamantinus* | Cropland | 14 | 6.00 | 3.97 |
|  |  | Caatinga remnant | 14 | 3.79 | 2.38 |
|  | *Jatropha mollissima* | Cropland | 14 | 11.36 | 7.81 |
|  |  | Caatinga remnant | 14 | 11.93 | 5.37 |
| High-quality interaction | *Cnidoscolus quercifolius* | Cropland | 14 | 2.36 | 1.75 |
|  |  | Caatinga remnant | 14 | 0.86 | 0.59 |
|  | *Croton adamantinus* | Cropland | 14 | 2.00 | 1.38 |
|  |  | Caatinga remnant | 14 | 0.36 | 0.43 |
|  | *Jatropha mollissima* | Cropland | 14 | 4.36 | 4.15 |
|  |  | Caatinga remnant | 14 | 2.57 | 1.66 |
| Low-quality interaction | *Cnidoscolus quercifolius* | Cropland | 14 | 3.14 | 2.77 |
|  |  | Caatinga remnant | 14 | 4.50 | 2.67 |
|  | *Croton adamantinus* | Cropland | 14 | 4.00 | 2.88 |
|  |  | Caatinga remnant | 14 | 3.43 | 2.11 |
|  | *Jatropha mollissima* | Cropland | 14 | 7.00 | 4.18 |
|  |  | Caatinga remnant | 14 | 9.36 | 5.19 |
| Seed removal | *Cnidoscolus quercifolius* | Cropland | 14 | 7.00 | 3.49 |
|  |  | Caatinga remnant | 14 | 0.71 | 0.57 |
|  | *Croton adamantinus* | Cropland | 14 | 6.79 | 4.72 |
|  |  | Caatinga remnant | 14 | 1.57 | 1.66 |
|  | *Jatropha mollissima* | Cropland | 14 | 1.93 | 1.05 |
|  |  | Caatinga remnant | 14 | 1.50 | 0.84 |
| Seed cleaning | *Cnidoscolus quercifolius* | Cropland | 14 | 0.90 | 0.08 |
|  |  | Caatinga remnant | 14 | 0.77 | 0.18 |
|  | *Croton adamantinus* | Cropland | 14 | 0.94 | 0.05 |
|  |  | Caatinga remnant | 13 | 0.89 | 0.11 |
|  | *Jatropha mollissima* | Cropland | 13 | 0.10 | 0.09 |
|  |  | Caatinga remnant | 13 | 0.04 | 0.03 |

**Figure S1.** Pearson correlation matrix of soil chemical and physical variables measured across 14 subsistence rainfed croplands and 14 adjacent Caatinga vegetation remnants. Variables include soil pH, aluminium (Al³⁺), hydrogen + aluminium (H + Al), phosphorus (P), nitrogen (N), sulfur (S), exchangeable bases (Ca²⁺, Mg²⁺, K⁺, Na⁺), sum of bases (SB), cation exchange capacity (CEC), and granulometric fractions (sand, silt, and clay). Colours represent the strength and direction of correlations (blue = positive; red = negative), with correlation coefficients shown in each cell.
